# Medical students’ career decision-making stress during clinical clerkships

**DOI:** 10.1007/s40037-022-00734-8

**Published:** 2022-12-07

**Authors:** Daan A. H. Fris, Annelies E. M. van Vianen, Jessie Koen, Matthijs de Hoog, Anne P. J. de Pagter

**Affiliations:** 1 Department of Pediatrics, Erasmus Medical Center—Sophia Children’s Hospital, Rotterdam, The Netherlands; 2 Work and Organizational Psychology, University of Amsterdam, Amsterdam, The Netherlands; 3 Department of Sustainable Productivity and Employability, Netherlands Organization for Applied Scientific Research (TNO), Leiden, The Netherlands; 4 Department of Pediatric Surgery & Pediatric Intensive Care Unit, Erasmus Medical Center—Sophia Children’s Hospital, Rotterdam, The Netherlands; 5 Department of Pediatric Hematology, Erasmus Medical Center—Sophia Children’s Hospital, Rotterdam, The Netherlands; 6 Department of Pediatrics, Leiden University Medical Center, Leiden, The Netherlands

**Keywords:** Career choice, Clinical clerkships, Career development, Career decision-making, Career decision-making stress, Specialty choice

## Abstract

**Objectives:**

Many medical students experience career decision-making stress in the final phase of training. Yet, the factors that induce or reduce career decision-making stress and how progression in their clerkships relates to these factors are unknown. This knowledge gap limits the possibilities for medical schools to develop and implement interventions targeting students’ career decision-making stress. This study explores content, process, and context factors that may affect career decision-making stress.

**Methods:**

Using cross-sectional survey data from medical master students (*n* = 507), we assessed content (future work self), process (choice irreversibility, time pressure, career decision-making self-efficacy), and context (supervisory support, medical school support, study load, competition) factors and their relationships with career decision-making stress. The hypothesized relationships were tested with structural equation modelling.

**Results:**

A clearer future work self and higher career decision self-efficacy were associated with lower career decision-making stress, while experienced time pressure, competition, and study load were associated with higher career decision-making stress. Choice-irreversibility beliefs, supervisory support, and medical school support were unrelated to career decision-making stress. As students’ clerkships progressed, they gained a clearer future work self, but also experienced more time pressure.

**Discussion:**

Clinical clerkships help students to form a clearer future work self, which can diminish career decision-making stress. Yet, students also experience more time pressure as the period of clerkships lengthens, which can increase career decision-making stress. A school climate of high competition and study load seems to foster career decision-making stress, while school support hardly seems effective in diminishing this stress.

**Supplementary Information:**

The online version of this article (10.1007/s40037-022-00734-8) contains supplementary material, which is available to authorized users.

## Introduction

Medical students experience significant stressors during clinical clerkships [1–7]. Recently, attention has been devoted to an underexposed yet prevalent stressor: career decision-making [2, 7]. Most medical students experience stress when having to make a career decision [8], with around 15% still undecided about their preferred career direction after graduation [9]. This is remarkable, because clinical clerkships not only help students gain work experience and practical skills, but should also provide them with an opportunity to explore career alternatives that enable them to make a career choice or alter their initial career preferences [10, 11]. As such, students’ initial concerns about having to make a career choice should gradually diminish as their clinical clerkships progress. The high prevalence of career decision-making stress, however, suggests that clinical clerkships are not always helpful in reducing this stress.

Extant research on the causes of career decision-making stress is fragmented and mostly based on qualitative methods [6, 12, 13]. Little is known about specific factors that influence career decision-making stress and how clerkship length is related to these factors. In this study, we aim to advance our understanding of what may influence career decision-making stress by integrating theory and research on careers in general with literature on career decision-making of medical students. Specifically, we combine the content-process-context (CPC) framework of career choice intervention [14] with previous findings on stressors during clerkships (i.e., study load [1, 2, 4] and competition [5]) to examine factors that are associated with career decision-making stress. The CPC framework includes content (e.g., clarity of future work aspirations), process (e.g., career decision-making self-efficacy), and context (e.g., competition) factors relevant to making career choices. The comprehensiveness of this framework lends itself especially well to obtain a complete picture of factors associated with career decision-making stress and the types of interventions that can alleviate it.

### Content factors

Content factors refer to elements of people’s self-concept regarding their work, including interests, abilities, values, needs and preferences [14]. People who have developed a well-defined self-concept with regard to their future work life and can link this to acceptable career roles tend to have less career decision-making stress [15]. Here, we examine medical students’ *future work self*, defined as a “representation of the future that reflects hopes and aspirations in relation to work” [16]. We expect that medical students’ future work self will be negatively related to career decision-making stress. The easier it is to envision a future work self, the less concerned one will be about having to make a career choice.

People develop their future work self by reflecting on their personal characteristics and possible career options [17]. Students, however, tend to have little work experience and a fragmented view of the labor market, which limits opportunities for self and career exploration. Internships can foster self-concept crystallization [18] and the exploration of work roles and environments that may fit the self [19]. Specifically, students can identify the working conditions they prefer or dislike [20]. Similarly, clinical clerkships may help medical students to gain a better understanding of the daily professional life of a clinician and trigger them to explore their own interests and capacities and the differences between medical specialties [12], which facilitate developing a future work self. We expect that the further along medical students are in their clerkships, the more developed their future work self will be.

### Process factors

Process factors influence the process of career decision-making, such as (dys)functional cognitions, cognitive biases, and heuristics [14]. Cognitions such as career decision self-efficacy, irreversibility beliefs, and time pressure may facilitate or impede career decision-making.

Career decision self-efficacy, i.e., the confidence one has in being able to successfully complete the tasks and behaviors required in making career decisions [21], is a crucial cognition facilitating the career decision process [22]. Individuals with high career decision self-efficacy tend to experience less career indecision [22], which is why we expect that medical students’ career decision self-efficacy will be negatively related to career decision-making stress.

Other cognitions may rather impede decision-making [23, 24]. One such dysfunctional cognition is the belief that a career decision is a once-in-a-lifetime and irreversible decision [23]. In general, people prefer reversible to irreversible decisions because they want to have optimal freedom of choice and avoid regretting their (possibly wrong) decision in the future [25, 26]. As such, the perception of an irreversible (career) choice may raise career decision-making stress in individuals [23]. Hence, we expect that choice irreversibility beliefs will be positively related to career decision-making stress.

Another dysfunctional cognition is time pressure, i.e., the experience of urgency of career decision-making. While high time pressure tends to promote action in people, for example when searching for a job, it is also associated with strain and lower mental health [27]. We therefore expect that time pressure will be positively related to career decision-making stress. Additionally, we expect that clerkship length will be positively related to experienced time pressure, because the distance to graduation and having to make a career choice becomes shorter as medical students progress through their clerkships.

### Context factors

Context factors are “key environmental features that can aid or impede choice-making and implementation” [14, p. 6]. Contextual supports, such as supervisory and medical school support, and contextual barriers, such as experienced competition and study load, can reduce and induce career decision-making stress, respectively.

Generally, supervisory support is an important resource in developing confidence in career decision-making [28, 29]. Also, academic career support services [30] and informational resources [22] can be useful in fostering confidence in career decision-making. We expect that supervisory support and medical school support will be negatively related to career decision-making stress.

As the demand for residency positions is higher than the supply, medical students may be uncertain if they can attain residency positions [1, 2]. This competition for residency places may make the selection of career goals more complicated and ambiguous, which may result in decisional stress. Further, medical students in the final stage of their training may experience a high study load [1–4], which can engender doubts about their fit to a profession and work environment that is characterized by a structural high workload [1]. We expect that competition and study load will be positively related to career decision-making stress.

## Methods

### Procedure

**Fig. 1:**
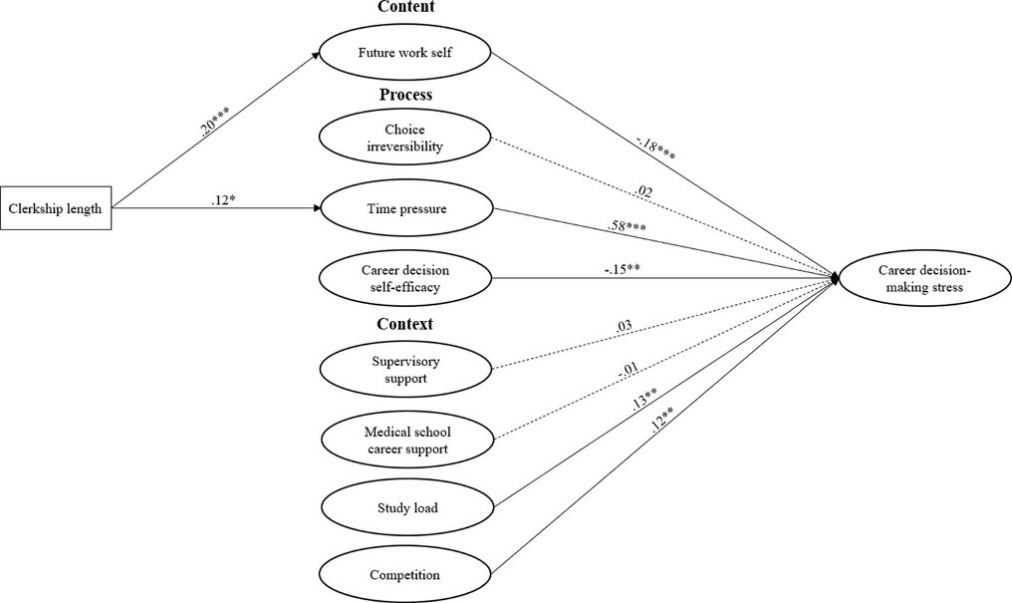
Hypothesized model and SEM results. Note: Dotted lines indicate non-significant relationships. ***p < 0.001 **p < 0.01 *p < 0.05 (2-tailed), controlled for gender differences

We tested our hypothesized model (Fig. 1) in a cross-sectional survey study, including validated measures, among medical master’s students from two medical schools, located in the north and west of the Netherlands. Following ethical approval from the institutional review board at the University of Amsterdam (IRB no. 2021-WOP-13139), study coordinators from both schools sent the students (*n* = 2293) an invitation by email to participate in a survey on career development. Participation was voluntary and anonymity was guaranteed. All participants gave informed consent. Participants who completed the survey received a digital gift card for an online store worth € 5.

### Measures

**Clerkship length** was measured by asking participants the number of weeks they had been doing their clerkships, with response options ranging from 0 to 100 weeks.

**Future work self** was measured with seven items based on the Future Work Self Salience scale [16]. Participants were first asked to describe their imagined future work, after which statements referring to their imagined work were presented. An example item is: “I can easily imagine my future work”. Items were rated on a scale ranging from 1 (strongly disagree) to 5 (strongly agree). The observed Cronbach’s alpha coefficient was 0.93.

**Career decision self-efficacy** was measured with three items from the goal selection subscale of the Career Decision Self-Efficacy Scale—Short Form [31]. An example item is: “I am able to determine what my ideal job would look like”. The items were rated on a scale ranging from 1 (strongly disagree) to 5 (strongly agree). The observed Cronbach’s alpha coefficient was 0.76.

**Career choice irreversibility** was measured using the three-item ‘criticality of the decision’ subscale from the Dysfunctional Career Decision-Making Beliefs Scale (DCB) [23]. A sample item is “Choosing a career is a crucial decision, so I must not make a mistake”. We added a reverse coded item (“My career choice during my master’s is a temporary choice, I can always change my career direction later”). Items were rated on a scale ranging from 1 (strongly disagree) to 7 (strongly agree). The observed Cronbach’s alpha coefficient was 0.81.

**Time pressure** was measured with four adapted items from the Job Search Time Pressure scale [27]. An example item is: “I have sufficient time to figure out what career direction fits me (R)”. Items were rated on a scale ranging from 1 (not applicable at all) to 5 (fully applicable). The observed Cronbach’s alpha coefficient was 0.86.

**Supervisory support** was measured with the six-item obtained social support scale [32]. An example item is: “My supervisors provide me with encouragement”. Items were rated on a scale ranging from 1 (strongly disagree) to 7 (strongly agree). The observed Cronbach’s alpha coefficient was 0.92.

**Medical school career support** was measured with four items that were drawn from the “lack of information about career alternatives” subscale of the CDDQ [33]. Items were adapted to reflect the extent to which the medical school provides career guidance and information about career options. An example item is: “My medical school provides enough information on different specialties”. Responses were rated on a scale ranging from 1 (not applicable at all) to 7 (fully applicable). The observed Cronbach’s alpha coefficient was 0.83.

**Study load** was measured with five adapted items from the Psychological Demands subscale of the Job Content Questionnaire (JCQ) [34]. An example item is: “My study requires me to work very hard”. Items were rated on a scale ranging from 1 (strongly disagree) to 5 (strongly agree). The observed Cronbach’s alpha coefficient was 0.85.

**Competition **was measured using the seven-item competition scale [35]. An example item is: “In my study there is an atmosphere of competition among students”. Items were rated on a scale ranging from 1 (not applicable at all) to 7 (fully applicable). The observed Cronbach’s alpha coefficient was 0.91.

**Career decision-making stress** was measured with four items from the Perceived Stress Scale [36]. The items were adapted to fit the current context. An example item is: “In the last four weeks, I felt nervous about important decisions I have to make regarding my future work”. Items were rated on a scale ranging from 1 (never) to 5 (often). The observed Cronbach’s alpha coefficient was 0.92.

**Control variables** Age, gender, medical school, parental profession and individual (career) guidance were included as control variables. Women tend to worry more than men [37] and age was found to be positively related to career decisiveness [38, 39]. We controlled for medical school to account for possible differences in career guidance programs and study culture, although there was no indication of a priori significant differences. Also, we asked students whether one or both of their parents work or have worked in a medical profession, 1 (*yes*) or 2 (*no*). Prior research has shown that medical students’ parents work in healthcare more often than the general population does [40] and parental profession is associated with career preferences of medical students [41]. In addition, parents working in healthcare may provide more adequate career support to their children as they have more knowledge of the work field. Finally, we asked students whether they received individual guidance from a study advisor, coach, student psychologist or mentor during their study, 1 (*yes*) or 2 (*no*). Research demonstrates that social support is an important resource in the career decision-making process [42, 43].

### Statistical analyses

Using MPlus 7.31, we first conducted a Confirmatory Factor Analysis (CFA) to evaluate the factor structure of the measures, that is, how well the items represent (i.e., loaded on) the hypothesized constructs after which we tested the hypothesized model using Structural Equation Modeling. We estimated relationship strengths using Full Information Maximum Likelihood. Future work self and time pressure were regressed on length in clerkships and career decision-making stress was regressed on the content, process, and context variables, which were allowed to covary. Additionally, we explored medical school and gender differences using MANOVAs and multigroup analyses.

## Results

### Sample

A total of 507 students responded to the survey (22.1%), 279 students from medical school A (response rate: 19.7%) and 228 from medical school B (response rate: 26.0%). This sample consisted of 73.8% (*n* = 374) female and 23.7% (*n* = 120) male participants (2.6% (*n* = 13) did not disclose their gender). The mean age was 24.3 years (*SD* = 2.28) and most participants had the Dutch nationality (*n* = 477, 94.1%). On average, participants were enrolled in the clinical clerkships for 34.11 weeks (*SD* = 23.64).

A power analysis revealed that our sample size (*n* = 507) was larger than the required sample size to detect effects (*n* = 460) [44]. Tab. 1 shows the means and standard deviations of the variables and the correlations between them.

**Table 1:** Means, standard deviations, and correlations

	*M* (*SD*)	1	2	3	4	5	6	7	8	9	10
1. Clerkship length	34.11 (23.64)	–									
2. Future work self	3.17 (0.82)	0.20**	–								
3. Choice irreversibility	4.21 (1.21)	−0.03	−0.08	–							
4. Time pressure	3.07 (0.91)	0.12*	−0.18**	0.38**	–						
5. CDM self-efficacy*	3.69 (0.65)	0.06	0.52**	−0.22**	−0.34**	–					
6. Supervisory support	4.48 (1.17)	−0.11*	0.06	0.03	−0.07	0.14**	–				
7. Medical school career support	3.58 (1.02)	−0.01	0.14**	−0.06	−0.14**	0.17**	0.35**	–			
8. Study load	3.26 (0.75)	0.03	−0.02	0.09	0.13**	−0.06	−0.21**	−0.07	–		
9. Competition	5.45 (0.96)	0.23**	0.11*	0.08	0.17**	0.06	−0.12*	−0.06	0.25**	–	
10. Career decision-making stress	3.13 (1.05)	0.16**	−0.32**	0.27**	0.61**	−0.38**	−0.09	−0.11*	0.23**	0.21**	–

**p < 0.01 *p < 0.05 (2-tailed); *career decision-making self-efficacy

### Confirmatory factor analysis

We used CFAs to examine the relationships between our observed variables and their underlying latent constructs. We compared a nine-factor model (i.e., a model in which the items of all variables included in the hypothesized model loaded on their respective factor) with a six-factor model (grouping conceptually similar process [choice irreversibility and time pressure] and contextual variables [supervisory support and medical school career support, competition and study load]) and a one-factor model (in which all items loaded on one factor). The nine-factor model yielded a reasonably good fit, χ2/df = 1.99, *p* < 0.001, TLI = 0.92; CFI = 0.93, RMSEA = 0.05, and fitted the data significantly better than the six-factor model, ∆χ2(21) = 1866.93, *p* < 0.001, or the common-factor model, ∆χ2(36) = 8144.47, *p* < 0.001 (see Table S1 in the Electronic Supplementary Material). These results provide support for our hypothesized factor structure.

The results of the hypothesized nine-factor model yielded the following ranges of standardized factor loadings (lowest to highest): future work self, 0.70–0.90; choice irreversibility, 0.55–0.93; time pressure, 0.61–0.84; career decision self-efficacy, 0.69–0.76; supervisory support, 0.77–0.84; medical school career support, 0.71–0.78; study load, 0.64–0.80; competition, 0.60–0.90, and career decision-making stress, 0.81–0.91.

### Hypotheses testing

The hypothesized model was tested with and without control variables. The results of these models were comparable. Because women experienced significantly higher career decision-making stress than men (*β* = 0.10, *p* = 0.01), gender was included in the further analyses. We omitted the other control variables to avoid an unnecessary decline in statistical power [45].

The hypothesized model showed an acceptable fit to the data, χ2/df = 2.17, *p* < 0.001, TLI = 0.90, CFI = 0.91, RMSEA = 0.05. Results (Fig. 1) showed that a clearer future work self (*β* = −0.18, *p* < 0.001) and higher career decision self-efficacy (*β* = −0.15, *p* = 0.007) were related to lower career decision-making stress. More time pressure (*β* = 0.58, *p* < 0.001), higher competition (*β* = 0.12, *p* = 0.004), and higher study load (*β* = 0.13, *p* = 0.004) were related to higher career decision-making stress. In contrast, choice irreversibility beliefs (*β* = 0.02, *p* = 0.65), supervisory support (*β* = 0.03, *p* = 0.54), and medical school support (*β* = −0.01, *p* = 0.80) were unrelated to career decision-making stress. Finally, clerkship length was associated with higher experienced time pressure (*β* = 0.12, *p* = 0.01) and a clearer future work self (*β* = 0.20, *p* < 0.001).

### Additional analyses

Because our participants were from two different medical schools that may differ in terms of educational culture, and because gender appeared to be a relevant control variable, we conducted additional analyses. We explored possible differences in variable means between medical schools and between men and women, and we tested equivalence among the patterns of relationships (Fig. 1). As an independent sample t‑test showed that clerkship length was significantly shorter for medical school A (*M* = 27.96, *SD* = 22.42) than for medical school B (*M* = 41.9, *SD* = 22.88), t(478) = −6.70, *p* < 0.001, we included clerkship length as covariate in the analyses.

#### Medical schools

A MANOVA (Tab. 2) yielded a statistically significant difference between the two medical schools, *F*(9, 410) = 9.78, *p* < 0.001, Wilks’ Λ = 0.82, partial *η*^*2*^ = 0.18. Further results show that participants from medical school A reported significantly higher supervisory support (*M* = 4.79, *SD* = 1.05) and lower study load (*M* = 3.13, *SD* = 0.75) and competition (*M* = 5.14, *SD* = 0.95) than participants from medical school B (*M* = 4.09, *SD* = 1.19, *M* = 3.45, *SD* = 0.72, *M* = 5.83, *SD* = 0.83, respectively) (see Table S2 in Electronic Supplementary Material). No significant differences were found regarding the other variables.

**Table 2:** Results of two MANOVAs testing medical school and gender differences^a^

	Medical schools						Gender					
Dependent variable	Sum of squares	*Df*	Mean Square	*F*	*P*	Partial η2	Sum of squares	*Df*	Mean Square	*F*	*p*	Partial η2
Future work self	0.12	1	0.12	0.18	0.67	< 0.001	1.37	1	1.37	2.13	0.15	< 0.01
Career choice irreversibility	0.31	1	0.31	0.21	0.65	< 0.001	2.12	1	2.12	1.45	0.23	< 0.01
Time pressure	0.07	1	0.07	0.09	0.76	< 0.001	0.31	1	0.31	0.38	0.54	< 0.001
Career decision-making self-efficacy	0.34	1	0.34	0.83	0.36	< 0.01	0.04	1	0.04	0.09	0.76	< 0.001
Supervisory support	44.16	1	44.16	35.37	< 0.001	0.08	0.31	1	0.31	0.23	0.64	< 0.001
Medical school career support	1.61	1	1.61	1.60	0.21	< 0.01	0.57	1	0.57	0.56	0.45	< 0.01
Study load	10.41	1	10.41	18.98	< 0.001	0.04	2.82	1	2.82	4.97	0.03	0.01
Competition	34.31	1	34.31	43.65	< 0.001	0.09	0.13	1	0.13	0.15	0.69	< 0.001
Career decision-making stress	0.77	1	0.77	0.73	0.39	< 0.01	4.39	1	4.39	4.17	0.04	0.01

^a^Length in clerkships was included as covariate

We did a multigroup analysis using Wald tests of parameter constraints to explore differences in relationship strength. To test overall differences in relationship strength between medical school A and B, all model parameters were constrained to be equal. Results showed no significant differences between the two medical schools in the overall model, ∆χ2(11, 480) = 14.84, *p* *=* 0.19. Yet, we tested whether the regression coefficients of two single parameters that appeared to differ between the medical schools were statistically different i.e., the relationship between length of clerkships and future work self (medical school A: *β* = 0.27, *p* < 0.001; medical school B: *β* = 0.09, *p* = 0.21) and the relationship between future work self and career decision-making stress (medical school A: *β* = −0.27, *p* < 0.001; medical school B: *β* = −0.05, *p* = 0.52). Again, multigroup analyses were done with only constraining the parameters of specific interest to be equal between the two medical schools. The regression coefficients of both relationships differed significantly (∆χ2(1, 480) = 3.86, *p* *=* 0.0496 and ∆χ2(1, 480) = 4.70, *p* *=* 0*.*03, respectively), meaning that length of clerkships was positively related to future work self and that future work self was negatively related to career decision-making stress in medical school A, whereas these relationships were absent in medical school B.

#### Gender

A MANOVA (Tab. 2) yielded statistically significant gender differences, *F*(9, 409) = 1.92, *p* = 0.048, Wilks’ Λ = 0.96, partial *η*^*2*^ = 0.04. Women reported significantly higher study load (*M* = 3.32, *SD* = 0.74) and career decision-making stress (*M* = 3.16, *SD* = 1.02) than men (*M* = 3.13, *SD* = 0.78, *M* = 2.9, *SD* = 1.1, respectively) (see Table S2 in the Electronic Supplementary Material). There were no gender differences in the other variables.

A multigroup analysis demonstrated no significant gender differences in regression coefficients in the overall model, ∆χ2(10, 472) = 12.92, *p* *=* 0.23. Yet, we tested the regression coefficients of two single parameters indicating gender differences separately (i.e., the relationship between supervisory support and career decision-making stress and the relationship between competition and career decision-making stress were constrained to be equal for men and women). There are gender differences if the Wald test of parameter constraints is significant. The relationship between supervisory support and career decision-making stress, ∆χ2(1, 472) = 6.47, *p* *=* 0.01, differed for men (*β* = −0.19, *p* = 0.05) and women (*β* = 0.10, *p* = 0.07). The relationship between competition and career decision-making stress did not significantly differ for men (*β* = 0.01, *p* = 0.95) and women (*β* = 0.15, *p* < 0.01), ∆χ2(1, 472) = 1.83, *p* *=* 0.18.

## Discussion

Choosing a career trajectory is one of the most prevalent stressors for medical students during their clinical clerkships [2, 7]. Based on the content-process-context (CPC) framework of career decision-making [14], we examined content, process, and context factors that could influence career decision-making stress during clinical clerkships. Our results demonstrated that as students’ clerkships progressed, they gained a clearer picture of their preferred future as a medical professional, which was negatively associated with career decision-making stress. However, as the end of their clerkships approached, students also experienced more time pressure to make a career decision, which was positively associated with career decision-making stress. We further uncovered that higher career decision-making self-efficacy, lower study load, and lower experienced competition were associated with lower career decision-making stress, but also that neither supervisory nor medical school support could help to diminish this stress.

### Findings and implications

Our findings highlight the essential role that clerkships play in the career development of medical students. Clerkships allow students to gradually form a better image of their hopes and aspirations in becoming a physician in a given field, which can diminish stress about having to make a career decision at the end of the clerkships. Yet, there is a flip side: as the clerkships progress, students tend to experience more time pressure, which is strongly associated with higher career decision-making stress. These findings suggest that medical students especially need support in coping with increased time pressure during their clinical clerkships.

Our study identifies some additional targets for intervention. To support the career decision-making process of students, medical schools could aim to enhance students’ career decision self-efficacy. Aligning with prior meta-analytical findings [22], our findings suggest that students who feel more efficacious to make a career decision experience less decisional stress. In addition, prior studies found that the contextual factors study load [1, 2, 4] and competition [5] were positively related to stress among medical students. This study adds to this extant research by demonstrating that these factors also relate to career decision-making stress. Our findings and those of prior research may urge medical schools to put effort in reducing study load and student competition.

Our finding that support from the supervisor and the medical school is not associated with career decision-making stress is surprising, because prior studies have demonstrated that supervisory support [28, 29] and academic support [30] are important resources in career decision-making. We suggest several explanations as to why our findings differ from those in prior research. First, the type of career support provided by the medical schools in this study may not have met students’ idiosyncratic needs [8, 46, 47] and may therefore not have been effective in reducing career decision-making stress. Second, supervisory support during clerkships may not always help alleviate career decision-making stress. Prior research [13, 48] has shown that supervisors do not always respond adequately to a student’s disclosed aspirations, especially if they do not sympathize with these aspirations. For example, studies by Woolley et al. [13, 48], demonstrate that supervisors may respond with negative treatment and discouragement, making students reluctant to express their aspirations. Third, we asked students to assess supervisory support in general, rather than support from a specific supervisor. Our measure may have been too crude to reveal potential positive effects, given that medical students have different supervisors for each clerkship who may differ in the degree and quality of their support.

Finally, we found differences between the respondents from the two medical schools regarding study load, competition, and supervisory support. In line with prior research [49, 50], these differences signal that study culture can differ between medical schools. Moreover, our results are consistent with previous research demonstrating that study culture affects student well-being [5], as we found that the more competition students perceived, the greater the career decision-making stress they experienced. Finally, we found that clerkship length was more strongly related to students’ future work self and that future work self was more strongly related to career decision-making stress in medical school A. Possibly, the clerkships in medical school A were more helpful in developing a clearer picture of one’s future than those in medical school B. Note, however, that students from medical school A were in their clerkships for a relatively shorter period of time than students from medical school B. During the starting phase of the clerkships, students may experience relatively more freedom of choice, allowing them to reflect on their own needs and aspirations, as compared to later stages when they become more aware of possible career constraints. Medical schools could pay specific attention to stimulating reflection in this early phase. Furthermore, as the clinical clerkships lengthen, medical schools could focus particularly on helping students to cope with time constraints and competition, both of which lead to uncertainty about being able to achieve future career plans. As such, it is worthwhile to explore in more detail which interventions at which stage of the clinical clerkship are effective in facilitating students’ career decision-making.

### Strengths and limitations

An important strength of this study is the quantitative and parsimonious approach used to investigate factors related to career decision-making stress during the clinical clerkships. The selection of factors was based on theory and career research, which amounted to a parsimonious, though profound, research model.

Still, some limitations should be considered when interpreting the findings of this study. First, the cross-sectional nature of this study does not allow conclusions to be drawn about the strength and direction of relationships. To further establish the direction of relationships and the effectiveness of interventions aimed at reducing career decision-making stress, a longitudinal design, experience sampling method, or randomized control trial is needed. It should be noted though, that we substantiated our hypothesized relationships with well-supported theories and prior research. Therefore, reverse causality of the proposed relationships seems unlikely. Second, common method bias [51] might have attenuated relationships between the variables in this study. We attempted to diminish the influence of common method bias by varying in response scales and guaranteeing response anonymity to participants [51]. Third, the data of this study were collected during the COVID-19 pandemic. It is possible that students experienced relatively more insecurity about setting and attaining their career goals [52] and their study progress might have been delayed [53]. Although the clinical clerkships occurred as normal during this study, future research is needed to replicate our findings under more normal conditions.

A fourth limitation of this study is its modest response rate, which could be caused by the recruitment method used. Students were invited by email and were not informed in class about the questionnaire. Other online survey research among Dutch medical students [8] showed a similar response rate (22.75%). Though we had no reason to believe that our results are biased, we tried to get an indication of the extent to which our sample reflects the population under study. To this purpose, we compared our sample with the population of medical students in medical school A and B and found that our sample did not differ substantially from the student population in terms of gender (sample: 74%, population: 68%) and age (sample mean: 24.3; population mean 25.5). All in all, we are confident that our sample is representative of the Dutch student population.

### Implications for medical education

The findings of this study bear a number of practical implications for medical education. This study provides medical schools with directives to develop targeted interventions to diminish career decision-making stress among medical students. First, medical schools could provide the tools (e.g., career counseling or coaching [42]) to stimulate the timely development of students’ future work self, preferably at an early stage of the clerkships when students can reflect on their own needs and aspirations unhindered by the constraints of the job market. Second, in later stages of the clinical clerkships, medical schools could focus on helping students cope with time pressure and competition, both of which promote career decision-making stress which reduces well-being and potentially fosters suboptimal decision-making [27]. Third, this study suggests that medical schools could invest in increasing the effectiveness of career supportive practices. For example, they could train supervisors to discuss career plans in an open and safe way and invest in developing informational resources on specialties and alternative career paths. Finally, medical schools could monitor students’ perceptions of the study culture and could involve students in developing interventions aimed at improving medical school culture.

## Conclusion

Medical students experience significant career decision-making stress during clinical clerkships. While the clerkships can be a helpful resource in the process of career decision-making, students can hardly draw upon additional resources that diminish decisional stress. Additionally, experienced time pressure and contextual factors such as study load and competition seem to increase career decision-making stress. Interventions aimed at facilitating students’ career decision-making could focus on developing a future work self early, strengthening students’ coping skills, and improving the study culture in medical schools.

## Supplementary Information

Table S1 contains information on the CFA’s of the hypothesized model and alternative models. Table S2 contains scale means, standard deviations, and sample sizes of medical schools, and males and females.
