# Supplementary material for: Medical students’ career decision-making stress during clinical clerkships
Source: Perspect Med Educ. 2022 Dec 7;11(6):350–8. doi: 10.1007/s40037-022-00734-8 (PMC9734734; doi:10.1007/s40037-022-00734-8)
Supplement: Supplementary file 1 — Table S1 contains information on the CFA’s of the hypothesized model and alternative models. Table S2 contains scale means, standard deviations, and sample sizes of medical schools, and males and females. [file 40037_2022_734_MOESM1_ESM.docx]

**Table S1.** CFA of hypothesized model compared to alternative models*.*

| Model | χ2 | d*f* | χ2/d*f* | *p* | | TLI | CFI | RMSEA | Model comparison | Δχ2 | Δd*f* | *p* |
| --- | --- | --- | --- | --- | --- | --- | --- | --- | --- | --- | --- | --- |
| 9-factor model^*^ | 1724.21 | 866 | 1.99 | <.001 | 0.92 | | 0.93 | 0.05 |  |  |  |  |
| 6-factor CPC model^†^ | 3591.14 | 887 | 4.05 | <.001 | 0.76 | | 0.77 | 0.08 | Model 1 > Model 2 | 1866.93 | 21 | <.001 |
| 1-factor model^‡^ | 9868.68 | 902 | 10.94 | <.001 | 0.21 | | 0.24 | 0.15 | model 1 > model 3 | 8144.47 | 36 | <.001 |

Note: We used the TLI (Tucker–Lewis index), CFI (comparative fit index), and RMSEA (root mean square error of approximation) estimates to evaluate model fit. Hu and Bentler (1999) [54] suggested TLI and CFI scores of close to .95 (and higher) for good model fit and RMSEA scores of .06 or lower. ^*^The items of all variables included in the hypothesized model load on their respective factor; ^†^process, content, and context domains are treated as latent factors; ^‡^all items load on one factor.

**Table S2*.*** Scale means, standard deviations, and sample sizes of medical schools, and males and females.

|  | Medical school | *M* | *SD* | *n* | Gender | *M* | *SD* | *n* |
| --- | --- | --- | --- | --- | --- | --- | --- | --- |
| Future work self | A. | 3.14 | 0.84 | 234 | Male | 3.27 | 0.74 | 99 |
|  | B. | 3.21 | 0.79 | 187 | Female | 3.15 | 0.84 | 321 |
|  | Total | 3.18 | 0.82 | 421 | Total | 3.17 | 0.82 | 420 |
| Career choice irreversibility | A. | 4.19 | 1.19 | 234 | Male | 4.33 | 1.20 | 99 |
|  | B. | 4.21 | 1.24 | 187 | Female | 4.16 | 1.21 | 321 |
|  | Total | 4.20 | 1.21 | 421 | Total | 4.20 | 1.21 | 420 |
| Time pressure | A. | 3.02 | 0.89 | 234 | Male | 3.11 | 0.81 | 99 |
|  | B. | 3.12 | 0.93 | 187 | Female | 3.05 | 0.93 | 321 |
|  | Total | 3.06 | 0.90 | 421 | Total | 3.06 | 0.91 | 420 |
| Career decision-making self- | A. | 3.66 | 0.65 | 234 | Male | 3.72 | 0.64 | 99 |
| efficacy | B. | 3.75 | 0.65 | 187 | Female | 3.70 | 0.65 | 321 |
|  | Total | 3.70 | 0.65 | 421 | Total | 3.70 | 0.64 | 420 |
| Supervisory support | **A.** | **4.79** | 1.05 | 234 | Male | 4.54 | 1.17 | 99 |
|  | **B.** | **4.09** | 1.19 | 187 | Female | 4.46 | 1.17 | 321 |
|  | Total | 4.48 | 1.17 | 421 | Total | 4.48 | 1.17 | 420 |
| Medical school career | A. | 3.63 | 1.00 | 234 | Male | 3.51 | 1.11 | 99 |
| support | B. | 3.51 | 1.00 | 187 | Female | 3.59 | 0.97 | 321 |
|  | Total | 3.57 | 1.00 | 421 | Total | 3.57 | 1.00 | 420 |
| Study load | **A.** | **3.13** | 0.75 | 234 | **Male** | **3.13** | 0.78 | 99 |
|  | **B.** | **3.45** | 0.72 | 187 | **Female** | **3.32** | 0.74 | 321 |
|  | Total | 3.27 | 0.76 | 421 | Total | 3.28 | 0.76 | 420 |
| Competition | **A.** | **5.14** | 0.95 | 234 | Male | 5.47 | 0.94 | 99 |
|  | **B.** | **5.83** | 0.83 | 187 | Female | 5.44 | 0.97 | 321 |
|  | Total | 5.45 | 0.96 | 421 | Total | 5.45 | 0.96 | 420 |
| Career decision-making | A. | 3.00 | 1.06 | 234 | **Male** | **2.90** | 1.10 | 99 |
| stress | B. | 3.21 | 1.01 | 187 | **Female** | **3.16** | 1.02 | 321 |
|  | Total | 3.09 | 1.05 | 421 | Total | 3.10 | 1.05 | 420 |

Note: Significant differences (see Table 2) between medical schools (A vs. B) and gender (male vs female) are in bold.
